# Yet another journal …

**DOI:** 10.4103/0972-9941.15238

**Published:** 2005-03

**Authors:** Tehemton E. Udwadia

**Affiliations:** Emeritus Professor of Surgery, Grant Medical College and J J Hospital, Consultant Surgeon, Head, Department of Minimal Access Surgery, P D Hinduja National Hospital, President, International Federation of Societies of Endoscopic Surgeons

Is there any rationale or even requirement for yet another journal devoted to Minimal Access Surgery (MAS)? MAS is snowballing with the volume and speed of an avalanche and is already well served by excellent, established Journals whose monthly contribution to MAS is so diverse and extensive, that any surgeon would be hard-pressed to go through all that is already being written on this subject. Why then the need for this Journal of Minimal Access Surgery?

The Asia-Pacific region is a vast ocean of humanity, which after decades is reaching for its economic and political potential. In similar vein surgery in this vast area is taking giant steps forward and these steps are best seen in the phenomenal growth of MAS When the first hesitant steps of laparoscopic surgery were taken in India in early 1990, the first in the developing world, “surgical activists” came down heavily on proponents of this patient-friendly surgery on grounds that it was “inappropriate” for poor countries, was a surrender to the West, would distort the values of surgeons in India and bring into question the ethics of those who promote this surgery.[1,2,3] The phenomenal growth of MAS throughout the Asia-Pacific region and its spread to small towns and the less privileged is a fitting response to those who would thwart surgical progress on grounds of poverty. There can be no doubt that vast discrepancies exist in the scope and quality of surgical care in all developing countries and great efforts need to be made to rectify the enormous gap between the rich and the poor, economically as also surgically,[4] but if surgery is a humanitarian science it should be our unrelanting endeavour to spread the benefits of surgical advance to all people in all places.[5] This then is the rationale for the JMAS. Having witnessed over the last fifteen years the phenomenal growth, vast numbers of patients treated and the incredibly impressive quality of MAS all over the Asia-Pacific region, the Indian Association of Gastrointestinal Endo-Surgeons felt that this volume and quality of MAS, as specifically practised in an economically deprived mileu, needed documentation, dissemination and should be “show-cased” in its own Jounral.

The flame of MAS was first lit in the developed world. The U.S. and countries in Europe have greatly helped nurture and spread the growth of MAS in poor countries by spreading education and funding equipment in the early years. Perhaps more, these countries have set a bench-mark in standards for self-appraisal and evaluation, in the quality of their surgical literature and in the leadership and thrust their two major Societies, the Society of American Gastrointestinal Endoscopic Surgeons [SAGES] and later the European Association for Endoscopic Surgery [EAES] have had on the growth and quality of MAS world wide. It is the anticipation of the JMAS that these countries will continue their support to the developing world as also to JMAS with their quality articles, input and suggestions, an anticipation well reflected by the composition of this Editorial Board.

However, the raison d’etre of this Journal is primarily to give scientific expression to the work done in this region as also other developing countries as also in Latin America and Africa. It is a sad fact that perhaps as a result of the pressure of the large volume of day to day work, or a lack of appreciation of their own quality and results the vast majority of surgeons here do not give structured, statistically significant expression to their surgical work. Just as fifteen years back we accepted the challenge to spread MAS in our part of the world, we now urge the vast army of surgeons active in MAS in all developing countries to give substance and expression to our extensive patient load and experience, share our cost-saving expertise, our innumberable innovations born out of stark necessity, our problems and solutions, and by encouraging more and more hesitant colleagues, help spread our mission to all corners.

One reads in MAS literature articles devoted to successful outcomes and positive evaluation of procedures. Rarely does one read of mishaps or complications, these are so conveniently swept under the carpet. Usually these unfortunate situations are isolated cases but if reported would very possibly be of greater practical and educational value than many of the success stories. It is the the intent of this Journal to publish as many such reports / papers and give total encouragement to those brave and positively motivated surgeons who have the courage and integrity to share their problem cases.

The world is fast becoming smaller and countries and continents rapidly coming closer. The International Federation Of Societies Of Endoscopic Surgeons (IFSES), a Federation of ten large Socieites of endoscopic surgeons which work in unision to supplement each other and help improve the quality and spread of MAS has four of its Member Societies in the Asia Pacific Region – the Japan Society for Endoscopic Surgery [JSES], the Society of Endoscopic and Laparoscopic Surgeons of Asia [ELSA], the Chinese Society for Laparo-Endoscopic Surgery [CSLES], the Indian Association of Gastrointestinal Endo-Surgeons[IAGES], and two in Latin America the Federation Latinoamericana de Cirugia[FELAC], the Association Latinoamaericana de Cirujanos Endoscopistas [ALACE]. The IAGES stretches out in support and co-operation to all these Societies, welcomes their contributions to JMAS. Most of these Societies have had and continue to have the support of the two major Societies of the developed world, the Society of American Gastrointestinal Endoscopic Surgeons and European Association for Endoscopic Surgery in their bid to narrow the gap between the priviledged and the deprived.

The Editorial Board is fully aware of the hurdles and problems it faces in establishing and indexing this Journal. On the other hand this Editorial Board stands committed to its conviction that this Journal will be an important vehicle in improving the quality and spread of MAS in this region. In this cause we confidently anticipate the total support not only of the entire surgical community in the Asia-Pacific region but from all over the world. If success is defined not so much by the position reached, as by the determination to overcome obstacles while trying to succeed, this Journal has charted its road-map in the correct direction.

## References

[1] Cheslyn-Curtis S (1991). Laparoscopic cholecystectomy. Natl Med J India.

[2] Antia NH (1994). Keyhole surgery. Lancet.

[3] Banerjee JK (1995). Keyhole surgery. Natl Med J India.

[4] Udwadia TE (2003). Surgical care for the poor – a personal indian perspective. Indian J Surg.

[5] Udwadia TE (2001). “One world–one people–one surgery”. Surg Endosc.

